# Methodological considerations in the analysis of contextual determinants of tuberculosis mortality—authors’ response

**DOI:** 10.1016/j.lana.2026.101690

**Published:** 2026-11-04

**Authors:** Yan Mathias Alves, Reginaldo Bazon Vaz Tavares, Bruno de Bezerril Andrade, Ricardo Alexandre Arcêncio

**Affiliations:** a Department of Maternal-Infant and Public Health Nursing, Ribeirão Preto College of Nursing, University of São Paulo, Ribeirão Preto, Brazil; b Division of Infectious Diseases, Department of Medicine, Johns Hopkins University, Baltimore, MD, USA; c Department of International Health, Bloomberg School of Public Health, Johns Hopkins University, Baltimore, MD, USA; d Clinical and Translational Research Laboratory, Gonçalo Moniz Institute, Oswaldo Cruz Foundation, Salvador, Bahia, Brazil

We appreciate Ma and Shan's^1^ comments on our ecological analysis of tuberculosis (TB) mortality across São Paulo municipalities^2^ and the opportunity to clarify methodological concerns.

First, they argued that omitting HIV/AIDS-related variables could result in residual confounding and overstate the contribution of socioeconomic factors in the model. To assess whether HIV burden influenced our findings, we incorporated municipal HIV detection rates in a sensitivity analysis restricted to 2020–2023 because of data availability. Estimates for socioeconomic covariates changed slightly, and spatial effects remained consistent (r = 0.999). However, because detection rates capture diagnosed cases rather than underlying burden, residual confounding by HIV cannot be excluded.

Second, they questioned whether Primary Health Care (PHC) coverage values exceeding 100% represented service utilization rather than population coverage. We clarify that the Brazilian Ministry of Health indicator measures potential PHC capacity, based on team numbers, types, and estimated capacity relative to the municipal population.^3^ This indicator reflects potential municipal service capacity rather than individual coverage^3^, ^4^; values may therefore exceed 100% when team capacity exceeds the municipal population; international comparisons require caution.

Third, they argued that COVID-19 incidence, a potential contextual confounder, should not have been excluded solely through GAIC stepwise selection and that the positive temporal trend could reflect pandemic-related disruptions. We agree that a relevant confounder should not be excluded solely by statistical selection. COVID-19 incidence was therefore forced into the model for March 2020–December 2024; the magnitude and direction of the socioeconomic estimates and the positive temporal trend remained essentially unchanged, and model fit did not improve. These findings indicate that estimates remained stable after adjustment for COVID-19 incidence, although this indicator may not fully capture pandemic-related disruptions to health services.

Fourth, they suggested that the inverse association between lower educational attainment and TB mortality might reflect residual confounding or collinearity with the São Paulo Municipal Development Index. We conducted a sensitivity analysis excluding the São Paulo Municipal Development Index (IPDM) from the model; the inverse association with adults aged ≥18 years without complete elementary education remained statistically significant and in the same direction. Replacing this variable with incomplete secondary education produced the same direction of association, although with poorer fit. Removing either the IPDM or any of the variables tested for educational attainment worsened model fit, suggesting complementary contextual information. The educational indicators were highly correlated (r = 0.93), and their simultaneous inclusion markedly changed coefficients, supporting the use of one educational indicator in the main model.

Given the ecological design, all associations are contextual. In particular, the educational attainment finding does not indicate an individual protective effect of lower education, and none of the associations should be interpreted causally. We thank the authors for helping to strengthen the interpretation of our findings.

## Contributors

YMA and RBVT contributed equally to data analysis, conceptualization of the Correspondence, and drafting of the original manuscript. BBA and RAA critically revised the manuscript for important intellectual content. All authors approved the final version of the manuscript.

## Availability of data and materials

The data used in this study are derived from routinely collected health information systems and are available from the corresponding author upon reasonable request, subject to applicable data protection regulations.

## Declaration of interests

The authors declare no competing interests.
